# Nanopesticides and Nanofertilizers: Emerging Contaminants or Opportunities for Risk Mitigation?

**DOI:** 10.3389/fchem.2015.00064

**Published:** 2015-11-16

**Authors:** Melanie Kah

**Affiliations:** Department of Environmental Geosciences, University of ViennaVienna, Austria

**Keywords:** nanotechnology, plant protection product, risk, environment, agriculture, nanoformulation, agrochemical

## Abstract

Research into nanotechnology applications for use in agriculture has become increasingly popular over the past decade, with a particular interest in developing novel nanoagrochemicals in the form of so-called “nanopesticides” and “nanofertilizers.” In view of the extensive body of scientific literature available on the topic, many authors have foreseen a revolution in current agricultural practices. This perspective integrates scientific, regulatory, public and commercial viewpoints, and aims at critically evaluating progress made over the last decade. A number of key (and sometimes controversial) questions are addressed with the aim of identifying the products that will soon emerge on the market and analyzing how they can fit into current regulatory and commercial frameworks. Issues related to the differences in definitions and perceptions within different sectors are discussed, as well as our current ability to assess new risks and benefits relative to conventional products. Many nanoagrochemicals resemble products used currently, which raises the question whether the effect of formulation has been sufficiently taken into account when evaluating agrochemicals. This analysis identifies directions for future research and regulatory needs in order to encourage intelligent design and promote the development of more sustainable agrochemicals.

## Introduction

This perspective focuses on applications of nanotechnology for plant protection and nutrition, in the form of nanopesticides or nanofertilizers, later referred to as nanoagrochemicals. The use of agrochemicals is crucial to modern agriculture, but the development of nanopesticides and nanofertilizers has received less, or at least delayed attention relative to other sectors of the food chain, such as food processing or packaging. Due to their direct and intentional application in the environment, nanoagrochemicals may be regarded as particularly critical in terms of possible environmental impact, as they (would) represent the only intentional diffuse source of engineered nanoparticles in the environment (Kah et al., 2013).

The use of agrochemicals is associated with some risks for human and environmental health (e.g., contamination of water resources, residues on food products). Many reports foresee that nanotechnology will allow the development of high-tech agricultural fields, equipped with a range of intelligent nanotools that allow for the precise management and control of inputs, including pesticides, fertilizers, and water. The development of such devices would certainly lead to a revolution in agricultural practices, and could possibly contribute to an important reduction in the impact of modern agriculture on the environment and an improvement in both the quality and quantity of yields (Scott and Chen, 2002; ETC, 2004; Sekhon, 2014; Liu and Lal, 2015). However, because agriculture is a low profit industry, one must recognize that such applications do not fit within current economic reality and also face a high risk of early regulatory and social rejection.

**Figure 1:**
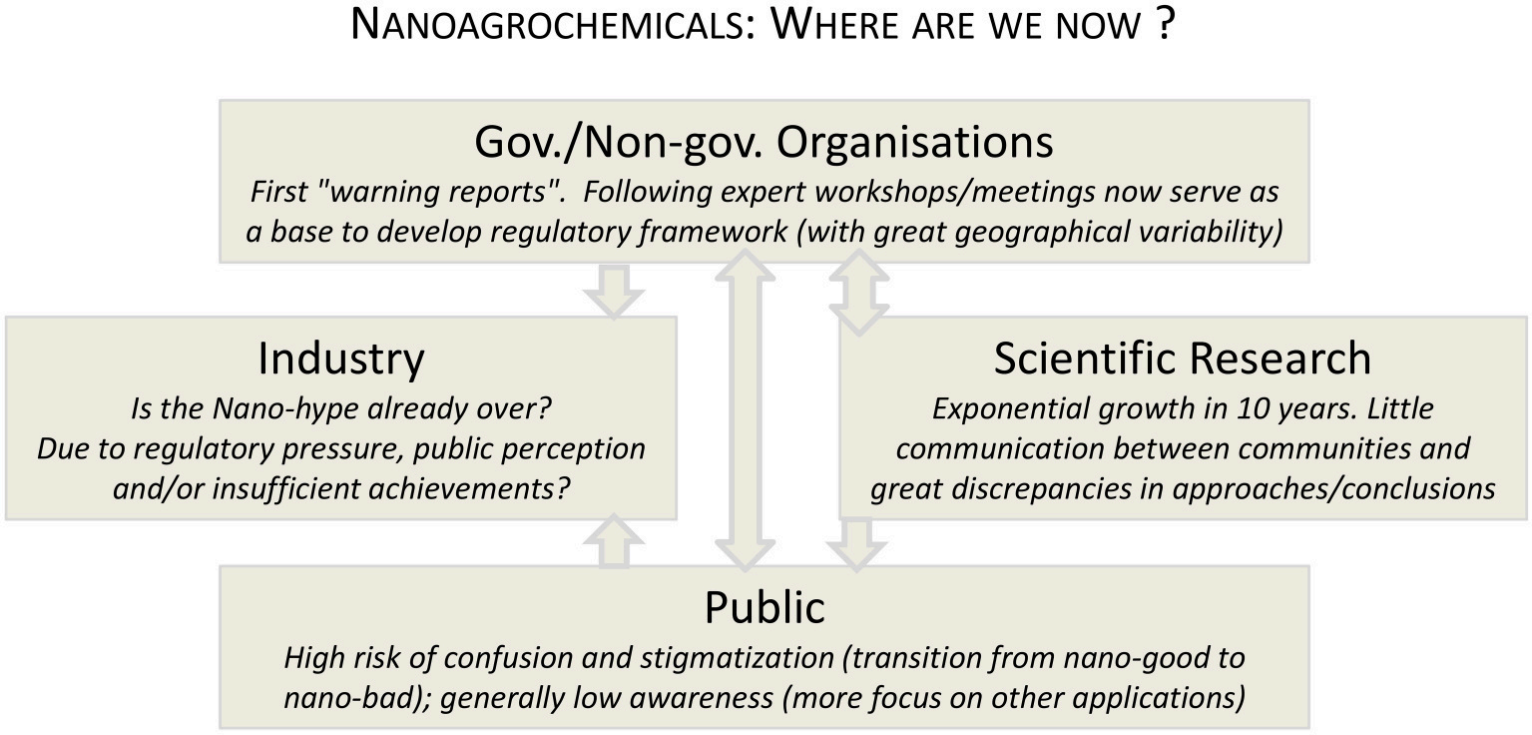
**Activities carried out over the last decade were intense, but fragmented by sectors, with only limited interactions (represented with the arrows) between the research sphere, governmental, and non-governmental organizations, industry, and the public**.

After briefly summarizing the activities related to nanoagrochemicals undertaken over the last decade (Figure 1), a number of key questions are addressed with the aim of identifying the products that may soon emerge on the market and analyzing how they fit into the current regulatory and commercial frameworks. Viewpoints from the scientific, industrial, and regulatory spheres are integrated to discuss what the future of nanoagrochemicals may look like. Finally, future directions are suggested that may allow the agrochemical sector to take advantage of nanotechnology, and possibly reduce its impact on human and environmental health.

## A decade of intense (but compartmented) activities in the research sphere

Scientific activities related to the development of nanopesticides and nanofertilisers have been remarkable and the number of peer-reviewed papers related to the topic has shown an exponential growth over the last decade. The different types of products presented in the literature and the latest trends in research have been regularly summarized (e.g., Gogos et al., 2012; Kah et al., 2013; Kah and Hofmann, 2014), though keeping updated is a difficult task now that several hundreds of papers are published on the topic each year. A total of 425 hits, for instance, are returned by searching the Web of Science, TOPIC = nano^*^ and (pesticide or fertilizer), only for 2014.

The popularity of the topic recently extended to major scientific meetings targeting various scientific communities (e.g., ACS, 2014; SETAC, 2014; Crop Chemical Europe, 2015). The trend is expected to continue, as the topic has recently been integrated as a research priority by various regulatory bodies and research funding agencies (e.g., USDA, 2015). There are huge differences in the research approaches applied to nanoagrochemicals in the different scientific communities involved. Some communities tend to convey a very positive image of the technology (e.g., formulation and material scientists), while others mainly communicate on the notion of risk (e.g., environmental scientists). The opinions presented by researchers can have a great impact on the perception that non-scientific communities develop.

## Governmental and non-governmental organizations

Several international organizations have coordinated workshops on the applications of nanotechnology for the agricultural sector, and conclusions were often compiled in reports that are available online (e.g., FAO/WHO, 2010, 2013; JRC-IPTS, 2014).

Activities by governments and regulatory bodies looking at developing pieces of legislation that are adapted to nanoagrochemicals that might emerge vary considerably (FAO/WHO, 2013; APVMA, 2014). The extent to which nanoagrochemicals develop will be strongly influenced by the regulatory system that controls their entry into the market. There are, at present, great geographical discrepancies, which may eventually shape applications emerging in a given market (Watson et al., 2011). In the EU for instance, some companies are currently facing great challenges derived from the definition of nanomaterials that has been proposed (EU, 2011). Companies are thus unlikely to choose the EU to introduce a new nanoagrochemical onto the market. In view of the general proliferation of nanoregulations worldwide, there is an urgent need for increased clarity in defining what constitutes a nanoagrochemical and harmonization of methods for assessing their risks. Only a few initiatives have been taken with this objective so far, (e.g., an expert workshop looking at facilitating a harmonized risk assessment of nanopesticides, whose conclusions were compiled in Kookana et al., 2014). Industry has a key role to play, for instance, by supplying the necessary data and product information, and sharing their technical, scientific and policy expertise (Watson et al., 2011).

## Industry

Knowledge about R&D in industry is limited by confidentiality issues, but while the “nanotechnology hype” continues steadily in other sectors, no clear applications seem to have emerged from the agrochemical industry so far. Ten years ago, expectations regarding nanotechnology were considerable and often associated with the names of big players: “Monsanto, Syngenta and BASF are developing pesticides enclosed in nanocapsules or made up of nanoparticles. The pesticides can be more easily taken up by plants if they're in nanoparticle form; they can also be programmed to be time-released” (Lauterwasser, 2005). Such statements were probably considered to contribute to a positive image of industry investing in promising R&D strategies. Similar declarations have now become scarce in geographical areas where the regulatory burden has greatly increased over the last decade (e.g., EU). The prefix “nano” may no longer be perceived positively and some companies seem to be distancing themselves from the technology. For instance, there are no more references to nanotechnology when searching the websites of large agrochemical companies (Suppan, 2013) while other companies have applied marketing strategies such as rebranding products or whole companies (e.g., ViveNano, Inc. changed their name to Vive Crop Protection, Vive Crop Protection, 2012).

## Public awareness

There are big concerns about the possible stigmatization of nanomaterials. A representative of the European Crop Protection Association explains: “In particular, the combination of nanotechnology, food and pesticides has a high potential of arousing public concern. The crop protection industry is afraid of the possibility of a scenario comparable with the rejection of genetically modified organisms” (JRC-IPTS, 2014). Despite a number of initiatives to warn the public that nanoparticles are now intentionally introduced at all stages of the food chain (e.g., Friends of the Earth, 2008), public awareness about nanoagrochemicals seems to remain generally low. This may be explained by (i) most concerns being focused on nanoparticles used as ingredients and additives to food and food packaging (e.g., TiO_2_, nanoAg), and (ii) inherent concerns associated with the use of agrochemicals (e.g., pesticides do not need to be associated with “nano” to be a topic of concern).

Overall, increasing regulatory burden and risk of stigmatization certainly played a role in the apparent decreased interest of the agrochemical industry in nanotechnology. However, the most important reason may be that research so far has not suggested that nanotechnology alone is likely to help with industry's research priorities, e.g., the discovery/elucidation of new modes of action. As explained by a representative of the European Crop Protection Association: “The nanosize so far did not demonstrate to hold important product changes of agrochemical interest. […] Agrochemical large companies are constantly exploring the possibilities offered by nanotechnology, among other innovative technologies” (JRC-IPTS, 2014).

## What is a “nanopesticide” or a “nanofertilizer?”

The terms “nanopesticide” and “nanofertilizer” have been extensively used, but sometimes with very different meanings according to the context. Inventories presented to date and based on patent analysis and scientific literature (e.g., Gogos et al., 2012; Kah et al., 2013) indicate that the terms can designate a very wide range of products regarding size, nature, level of development and even relevance for agricultural practices. In the scientific literature, the prefix “nano” has been associated until now with the notion of novelty and implicitly suggests superior properties relative to non-nano counterparts. Hence, many formulations were named “nano” with the main objective of increasing attention and possibly facilitating publication.

When the information makes its way to non-specialist readership (e.g., in press releases, interviews, reports), there is a risk of confusion about what a nanopesticide or a nanofertilizer is and how it relates, for instance, to the definitions that have been proposed for regulatory purposes.

## Are nanoagrochemicals considered “nano” by regulators?

Overall, the hypothesis that smaller means more reactive and, thus, more potent has not been substantiated for agrochemicals. The majority of nanopesticides described as “nano” in literature greatly exceed the 100 nm size boundary that has been recommended for regulatory purposes. There are considerable issues relating to the definition of nanoparticles and how the criteria proposed could apply to nanopesticides (discussed in Kah et al., 2013). Most importantly, a definition based on size alone would exclude many recent so-called nanoformulations and, on the other hand, include products that have been on the market for decades without posing particular problems (e.g., microemulsions, formulants such as clays and polymers). The EU initiative of a repository for nanomaterials (EC, 2014), therefore, comes with the risk to further confuse consumers by including ingredients that have been used for decades without previously being classified as “nano” (JRC-IPTS, 2014). In this context, it may be more useful to speak about nano-enabled or formulation technology, rather than focusing only on the nanoparticles and how they should be defined.

## Are nanopesticides on the market?

This recurrent question cannot be answered until a clear definition has been agreed on, which explains why contradictory statements have been made by members of different communities. Some representatives of governmental organizations have suggested that there are “no registered pesticides on the market which contain nanomaterials” (EFSA, 2009) and that “nano-enabled pesticides [are] still a dream at the moment” (S. Ramaswamy, Director of the USDA National Institute of Food and Agriculture, Gewin, 2015). Representatives of industry or research institutions have rather suggested that there are “very few, if any” or “not many” (JRC-IPTS, 2014). Finally, other groups have warned that “Manufactured nanoparticles, nano-emulsions and nano-capsules are now found in agricultural chemicals” (Friends of the Earth, 2008) or that “The first nano-formulations of pesticides are quietly making their way onto agricultural fields” (Gewin, 2015).

## Can the risks and benefits associated with the use of nanoagrochemicals be assessed?

When considering all nanoproducts that will possibly emerge in the food and agriculture sectors, there is a widely accepted consensus that there is insufficient reliable data currently to allow a clear safety assessment (FAO/WHO, 2013; JRC-IPTS, 2014).

When considering only nanoagrochemicals, the paradigm behind a classical risk assessment approach (i.e., hazard × exposure) is suitable, but applying approaches used within the current regulatory framework directly would result in a number of pitfalls (Kookana et al., 2014). Exposure assessment relies on investigations into the environmental fate of a compound. There have been a limited number of studies investigating nanoagrochemicals (Kah et al., 2013; Kah and Hofmann, 2014). It is also likely that fate and hazard endpoints are not adequately determined through the application of protocols that were developed previously for other types of chemicals (Kah et al., 2014; Kookana et al., 2014). Overall, the current level of knowledge appears to be largely insufficient for a reliable assessment of the risks associated with the use of nanoagrochemicals.

However, prohibiting the application of nanopesticides until they are proven entirely safe is unrealistic, as all pesticides are inherently toxic (at least to the target pest) and, thus, associated with some risk. It is also important to note that some nanopesticides may offer a number of benefits, including increased efficacy, reductions in application rates, exposure to non-target organisms or the development of resistances. In the scientific literature, the last couple of years have seen increasing incentives to use nanotechnology to develop products that may be less harmful to the environment relative to conventional agrochemicals.

A fair assessment of nanopesticides should, thus, be looking at evaluating both the risks and benefits associated with their use relative to current solutions. While this may not be possible when considering all products discussed so far in literature, restricting the analysis to products that are likely to emerge in the next decade shows that a fair assessment may be possible.

## Which types of nanoagrochemicals are ready to emerge in the next decade?

Many nanoagrochemicals described in the scientific literature do not fit within current market constraints. Many have low agronomic relevance, while others are associated with obviously unacceptable risks without clear benefits. For example, engineered nanoparticles that have received most of the attention in other sectors have very low potential for large-scale agricultural applications (e.g., carbon nanotubes, nanosilver). Similar to the trends observed in other sectors of the food chain over the last couple of years, interest has shifted from inorganic toward organic-based nanomaterials (e.g., nano-encapsulates, nano-composites, Aschberger et al., 2015). Nanoagrochemicals that use organic-based delivery systems developed for food or pharmaceutical applications are, however, often not economically competitive with other agrochemicals. More critical investigations assessing whether the products presented in the literature are able to compete with existing formulations in terms of both costs and performance are markedly needed.

Overall, nanoagrochemicals soon to emerge mostly consist of “nano” formulations of ingredients registered already and are, thus, very similar to many agrochemical products that are presently on the market (e.g., emulsions, suspensions).

## Are nanoformulations really new?

Development of new formulations has long been a very active field of research, since all agrochemicals need to be formulated for specific applications. Under increasing regulatory pressure, the application and delivery of authorized active substances need to be optimized more than ever before. Formulation scientists, thus, continue to explore new solutions aiming to enhance agrochemical activity, while, at the same time, keeping the environmental impacts to a minimum. In order to maintain colloidal stability and prevent phase separation during storage and application, most formulations contain structures belonging to the nanometer range (e.g., micelles) which also exist in many natural products (e.g., milk). Formulation scientists have now access to novel instruments that allow a better understanding of those structures, facilitating their synthesis and modifications to suit a given purpose.

“Nano-enabled” formulations could, for example, encompass those emulsions made of smaller micelles formed with smaller amount of surfactants, or microcapsules with a well-defined nanopore network. Such products have the potential to support a better management of agricultural inputs and, thus, to reduce the impact of modern agriculture. Hence, the regulation of a formulation on the market should not be solely based on a size threshold (i.e., above or below 100 nm), but rather rely on a science-based assessment of new risks and benefits involved, not only in terms of individual ingredients, but also based on how the whole formulation (i.e., active(s) and formulants) behaves in the environment.

## To what extent are the effects of formulation currently taken into account?

The effects of formulations on the environmental fate and effect of active substances in pesticides have been evaluated to a limited extent in the EU under Directive 91/414. The new EU regulation for pesticides (1107/2009) states that the impact of formulations should be taken into account, but it comes simultaneously with guidance documents suggesting that it is acceptable to assume “that formulants do not influence the fate and behavior of an active substance in the environment” (e.g., European Commission, 2009).

Pesticide authorization has long been subject to a strict and increasingly protective regulatory risk assessment. Safety factors are typically applied in order to account for uncertainties and provide a margin of safety. It is likely that the effects of formulations (nano or not) fall within this margin. This is probably the reason why a representative of the European Crop Protection Association considered that “under the current procedure for traditional crop protection products, the safety of nanomaterials would also be properly assessed” (JRC-IPTS, 2014), even though current scientific paradigms may not be adequate.

Using the highly conservative risk assessment strategy described above does not encourage the level of investment in R&D that is necessary to design formulations which reduce the risk (e.g., through reductions in application rates or spray drift). Impacts of (nano)formulations on the fate and effects of active substances have been reported on many occasions in the scientific literature, but the mechanisms involved remain poorly understood. Elucidating those processes and analyzing the consequences in terms of environmental impact requires the application of experimental protocols, analytical techniques and theories that are different to those typically applied to agrochemicals (e.g., colloidal chemistry). Kookana et al. (2014) discussed how combining and adapting the approaches developed for pesticides and nanoparticles could, in many cases, provide a reasonable assessment of the risks associated with nanopesticides. The same approach could also be successfully applied to assess the impacts of formulations likely to exhibit colloidal behavior upon application (independent from whether or not they were designated as “nano” according to criteria applied in the research, public or industry spheres).

## What may be the future of nanoagrochemicals?

**Figure 2:**
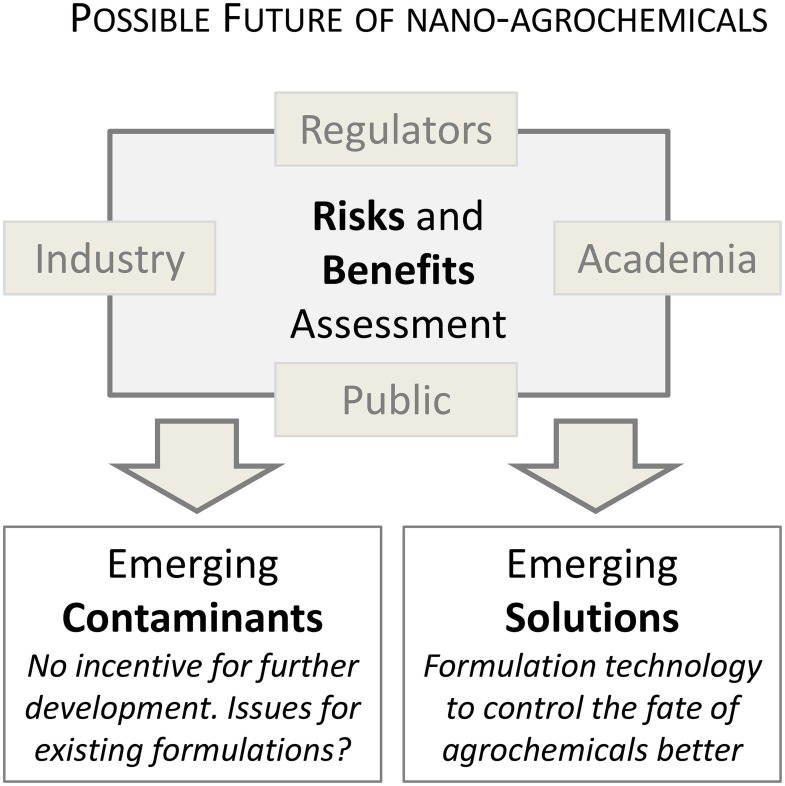
**The future of nanoagrochemicals may follow one of two scenarios**. In the first (most likely in the current context), nanoagrochemicals are considered as emerging contaminants and the development of the technology will remain limited. The second scenario will require the establishment of highly collaborative and interdisciplinary research to provide fair assessment of both risk and benefits so that the full potential of (nano)formulations can be explored.

Two potential scenarios that the development of nanoagrochemicals might follow in the future are illustrated in Figure 2. In the first, developments continue along the current path and nanoagrochemicals are likely to become, or at least be perceived as, the next emerging category of contaminants associated with agricultural practices. Alternatively, nanotechnology could become a potential source of emerging solutions to mitigate contamination by pesticides and fertilizers. This second scenario can only be achieved by rapid changes by industry, researchers, and regulatory agencies, following for instance, some of the directions suggested below.

- Available analyses of nanoagrochemicals typically consider all products discussed in literature, many of which represent an unacceptable risk, without being relevant for agricultural applications (e.g., ingredients that are extremely persistent or whose efficacy is marginal). Increasing collaborations between disciplines that are involved at all stages of the development and evaluation of agrochemicals (e.g., formulation, plant, material, and environmental scientists) will support the development of products fitting within the multiple constraints of the agrochemical sector and that are likely to bring an added value relative to existing products.
- The requirements of the latest EU directive regarding a better evaluation of formulations are typically viewed as additional constraints. Current approaches to chemical regulatory assessment often consist of applying incremental safety factors to account for the increasing level of uncertainty. Alternatively, new science-based tools should be developed to assess and fully exploit what the science of formulation has to offer, based on the risks and benefits over the entire life cycle of the products.
- With increasing regulatory pressure and risk of stigmatization, incentives are needed to promote innovation that may lead to the development of more intelligent solutions for plant protection and nutrition. Promotion of more collaboration across sectors (e.g., research, industry, and regulators) and integration of social science and law will ensure public/consumer acceptance and the development of suitable legal frameworks.
- Establishing a size threshold to distinguish “nano” from “non-nano” is of limited value for agrochemicals. Moving to a broader concept of nano-enabled technology and building on the experience from other sectors (e.g., food science, nanometrology) will be more valuable to support the development of more sustainable agrochemicals.

## Conflict of interest statement

The author declares that the research was conducted in the absence of any commercial or financial relationships that could be construed as a potential conflict of interest.
